# Dynamics of animal joint space use: a novel application of a time series approach

**DOI:** 10.1186/s40462-019-0183-3

**Published:** 2019-12-09

**Authors:** Justin T. French, Hsiao-Hsuan Wang, William E. Grant, John M. Tomeček

**Affiliations:** Department of Wildlife & Fisheries Science, Texas A&M University, 534 John Kimbrough Blvd., College Station, 77843 USA

**Keywords:** Bhattacharyya’s affinity, Beta distribution, Time series, Copula marginal regression, Joint space use, GPS telemetry, Utilization distribution

## Abstract

**Background:**

Animal use is a dynamic phenomenon, emerging from the movements of animals responding to a changing environment. Interactions between animals are reflected in patterns of joint space use, which are also dynamic. High frequency sampling associated with GPS telemetry provides detailed data that capture space use through time. However, common analyses treat joint space use as static over relatively long periods, masking potentially important changes. Furthermore, linking temporal variation in interactions to covariates remains cumbersome. We propose a novel method for analyzing the dynamics of joint space use that permits straightforward incorporation of covariates. This method builds upon tools commonly used by researchers, including kernel density estimators, utilization distribution intersection metrics, and extensions of linear models.

**Methods:**

We treat the intersection of the utilization distributions of two individuals as a time series. The series is linked to covariates using copula-based marginal beta regression, an alternative to generalized linear models. This approach accommodates temporal autocorrelation and the bounded nature of the response variable. Parameters are easily estimated with maximum likelihood and trend and error structures can be modeled separately. We demonstrate the approach by analyzing simulated data from two hypothetical individuals with known utilization distributions, as well as field data from two coyotes (*Canis latrans*) responding to appearance of a carrion resource in southern Texas.

**Results:**

Our analysis of simulated data indicated reasonably precise estimates of joint space use can be achieved with commonly used GPS sampling rates (*s*.*e*.=0.029 at 150 locations per interval). Our analysis of field data identified an increase in spatial interactions between the coyotes that persisted for the duration of the study, beyond the expected duration of the carrion resource. Our analysis also identified a period of increased spatial interactions before appearance of the resource, which would not have been identified by previous methods.

**Conclusions:**

We present a new approach to the analysis of joint space use through time, building upon tools commonly used by ecologists, that permits a new level of detail in the analysis of animal interactions. The results are easily interpretable and account for the nuances of bounded serial data in an elegant way.

## Background

Quantifying spatial overlap, or joint space, use between individual animals is of interest in many branches of ecology. How animals utilize space is a function of many factors, including resource availability [1], risk [2], and competition [3]. How these factors affect interactions between individuals is of key importance for many ecological issues. For example, joint space use has been linked to animal contact rates, and thus disease transmission [4, 5], animal social behavior [6, 7], as well as population genetics [8]. Though a common procedure, the analysis of joint space use remains problematic [9].

Ecologists commonly analyze space use in terms of an animal’s utilization distribution (hereafter UD), the 2-dimensional relative frequency (probability) distribution of animal locations in space [10]. UDs provide a continuous representation of the relative amount of time an animal spent at a given location, or the intensity of space use, facilitating easy interpretation. The probabilistic nature of UDs provides attractive properties that make them useful for home range estimation. For example, taking the isopleth contour at a given probability density can provide a demarcation of where an animal spent an arbitrary proportion of its time [10]. However, utilizing the parent distribution in further analyses permits deeper inference into the spatial interactions between individuals.

Quantifying the degree of joint space use between 2 individuals permits the testing of a variety of hypotheses about inter-individual interactions [11]. The 3-dimensional intersection of 2 UDs provides an estimate of spatial overlap that incorporates information about the relative intensity of space use by each individual. This provides a more robust estimate of joint space use compared to 2-dimensional approaches that use the shared area of UD isopleths. This joint volume can be measured using several indices, however Bhattacharyya’s Affinity (BA; [12]) has been shown to be minimally biased and has attractive properties that lend interpretability [11]. BA scales from 0 to 1, where 0 represents no spatial overlap and 1 represents identical space use. Theoretical bounds on behavioral metrics greatly facilitate ecological interpretation [13]. Several authors have utilized these pairwise comparisons to examine changes in joint space use between blocks of time (*sensu* [4, 14, 15]).

Though a common procedure in ecological literature, such an analysis oversimplifies temporal variation in joint space use. These interactions are dynamic in both time and space, making analysis of interactions inherently high-dimensional. Comparisons between few, relatively long time blocks provide limited insight into these processes, and overlook considerable temporal detail. Furthermore, they implicitly assume that animal space use patterns are stationary, or unchanging within the time period over which UDs are estimated [16]. This is unlikely to be the case for long periods of time, but such an assumption is much more reasonable over shorter intervals. Comparing UDs over finer, regular intervals (e.g. week or month) would reveal considerably more detail in patterns of spatial interactions, and permit statistical analysis of interaction dynamics, which was previously elusive [17].

We achieve such an analysis with a novel approach that synthesizes tools already familiar to ecologists and applies an appropriate regression framework. Abrahms et al. [18] derived a UD-based index of space use stability by measuring the intersections of successive monthly UD estimates for an individual. Though they did not consider trends in the sequence of measurements, their approach is readily extendable to examine dynamic interactions using a time series framework [17, 19], a logical avenue for the analysis of space use dynamics. When coupled, existing UD intersection metrics and time series analyses provide a simple, interpretable, and rigorously testable summary of complex dynamics of joint space use. This reduces a 5-dimensional problem (latitude, longitude, use intensity of 2 individuals, and time) to 2 manageable dimensions (spatial overlap and time). However, the bounded nature of BA precludes the use of standard regression procedures, such as normal linear regression or generalized linear models (GLMs). This is because GLMs are strictly suited to distributions with orthogonal (independent) parameters. The orthogonality assumption is violated when dispersion depends on the mean, which is a key property of bounded variables [20]. Other, analogous methods are needed to link the index to covariates.

Copula regression methods are a commonly used alternative to traditional GLMs in the financial and actuarial sectors [21] though, to our knowledge, their use in ecology is limited to one example [22]. They accommodate any response distribution, and are used to model complex correlation structures [23]. Recent work extends these methods to bounded time series [24], providing a link between the intersection index and explanatory variables.

Extending UD intersection metrics to a time series framework provides a flexible and interpretable approach to the analysis of space use interactions between individuals. Modeling joint space use in this way shows how the proportion of time 2 individuals use the same places changes through time, which is not only mathematically tractable, but intuitively understandable. This makes the results of our approach simple to communicate to both peers and non-scientists alike.

The success of this framework depends on the precision with which BA can be estimated with current GPS technology, which will affect both the sampling distribution of BA itself and the estimates of the effect of covariates on BA. Therefore, the goals of this work are: 1) To determine the precision with which BA could be estimated over reasonable sampling intensities; 2) to evaluate the accuracy and precision of effect size estimates of a covariate; and 3) to demonstrate the application of our methodology to a real data set. We simulated GPS data sets arising from known UDs at varying sampling intensities, then examined the precision of BA estimates from these simulations at high and low true values. We then evaluated the accuracy and precision of effect size estimates as sampling intensity increases. Finally, as an example, we examined the change in spatial interaction of 2 coyotes (*Canis latrans*) in southern Texas in response to a carrion deposition event.

## Material and methods

### Simulation study

We expanded simulation methods previously developed to evaluate kernel density estimator (KDE; [25]) performance as home range estimators [26, 27]. We used these simulations to a produce a known series of BA values with which we could compare estimates (Fig. 2). Each series consisted of 100 time windows (*t*). The true UD of each individual was held constant for the first half of the series, shifted to produce a known change in BA at *t*=50, and then held constant throughout the remainder of the series. We drew a specified number of locations randomly from the true UD of each individual at each time window, representing artificial GPS location data, to examine bias and precision as sampling intensity increases. By defining time periods a priori, we separate this analysis from home range estimation [27, 28]. In this context, an autocorrelated movement model would lead to an observed movement pattern that did not reflect the true UD on which we based our BA calculation. We sampled randomly from the true UD in order to ensure consistency between the within-window range and the location samples. We used simple bivariate normal (BVN) UDs with equal, unit variances with means separated by a fixed distance. We induced a 0.60 change in BA, from 0.20 to 0.80, at *t*=50 by changing the distances between means from 3.580 to 1.319.

We used a fixed KDE to fit a UD estimate for each individual at each time window. We used a bivariate normal kernel according to

1 $$ \widehat{UD_{it}} = \frac{1}{nh^{2}}\sum^{n}_{i-1}\frac{1}{2\pi} exp\left(\frac{-(\mathbf{x} - \mathbf{X}_{i})'(\mathbf{x} - \mathbf{X}_{i})}{2h^{2}}\right)  $$

where $\widehat {UD_{it}}$ is the estimated UD surface of animal *i* at time *t*, **x** is any location in 2-d space, **X**_*i*_ is the *i*^*th*^ observation of the animal’s location, *n* is the number of observations, and *h* is a smoothing parameter [25]. We used the reference smoothing parameter for computational simplicity, calculated as

2 $$ h = \sqrt{\frac{s^{2}_{x} + s^{2}_{y}}{2}}\cdot n^{-1/6}  $$

where $s^{2}_{x}$ and $s^{2}_{y}$ are the variances of the x and y coordinates, respectively [29].

We then calculated BA between the 2 simulated individuals at each time window to obtain a series of BA estimates,

3 $$ BA_{t} = \iint{\sqrt{\widehat{UD_{1t}}(x,y)}*\sqrt{\widehat{UD_{2t}} (x,y)} dx dy}  $$

where $\widehat {UD_{1t}}$ and $\widehat {UD_{2t}}$ are the UD estimates of individuals 1 and 2, respectively, at time *t*. We evaluated the bias and precision of BA estimates for sampling intensities of 50–1000 locations per temporal window, at increments of 50. We fit KDEs and calculated BA using the *adehabitatHR* package [30] in R [31].

We then evaluated how well we could estimate the effect size (magnitude of change) in BA due to our simulated disturbance at *t*=50. We used a marginal beta regression with a Gaussian copula [24] of the form

4 $$ \begin{aligned} Y_{t}|X \sim Beta(\mu_{t},\kappa_{t})\\ logit(\mu_{t}) = X^{\top}_{t}\beta \end{aligned}  $$

where *Y*_*t*_|*X* is the value of the BA series at time *t*, given covariates *X*, *μ*_*t*_ and *κ*_*t*_ are the mean and precision of the beta distribution at time *t*, respectively, and *β* is the vector of regression coefficients. Copula methods exploit the probability integral transformation to relate the beta distributed response *Y*_*t*_ to covariates *X*_*t*_,

5 $$ Y_{t} = F^{-1}_{t}\{\Phi(\epsilon_{t});\beta\}  $$

where *Y*_*t*_ is assumed to be marginally beta distributed, $F^{-1}_{t}\{\cdot ;\beta \}$ represents the appropriate cumulative density function linking the density to covariates (see [24]), and *Φ*(*ε*_*t*_) is the cumulative distribution function of the normal distribution with mean 0 and variance *ε*_*t*_. This allows the use of autoreggresive and moving average (*ARMA*(*p*,*q*)) terms, which are a special case of a multivariate normal covariance matrix [32], to model serial dependence in a non-Gaussian context [24]. The *ARMA*(*p*,*q*) term is defined as

6 $$ \epsilon_{t} = \sum^{p}_{i = 1}\psi_{i}\epsilon_{t-i} + \sum^{q}_{j = 1}\lambda_{j}\eta_{t-j} + \eta_{t}  $$

where *ε*_*t*−*i*_ is the error of the previous observation, *ψ*_*i*_ is an autoregressive parameter vector, *λ*_*j*_ is a moving average parameter vector, and *η*_*t*_ are independent zero-mean normal variables [24]. Parameters are estimated with maximum likelihood. The copula-based approach separates the linear predictor from the correlated error structure, meaning the regression coefficients are interpreted in the same manner as a GLM and not confounded by the *ARMA*(*p*,*q*) term. We refer interested readers to [24] for a detailed treatment on the role and advantages of copulas in the analysis of bounded time series.

We fit marginal beta regression models using a binary covariate corresponding to the known change in UDs at *t*=50 using the *gcmr* package [33] in R [31]. In ecological terms, this is analogous to estimating the effect of the presence of a resource, the implementation of some disturbance, a hypothesized season, or some other relevant binary variable, on the degree of spatial interaction between two individuals. We replicated the entire process 100 times for each level of sampling intensity to obtain the sampling distribution of our effect size as a function of sampling intensity.

### Application to empirical data

**Fig. 1:**
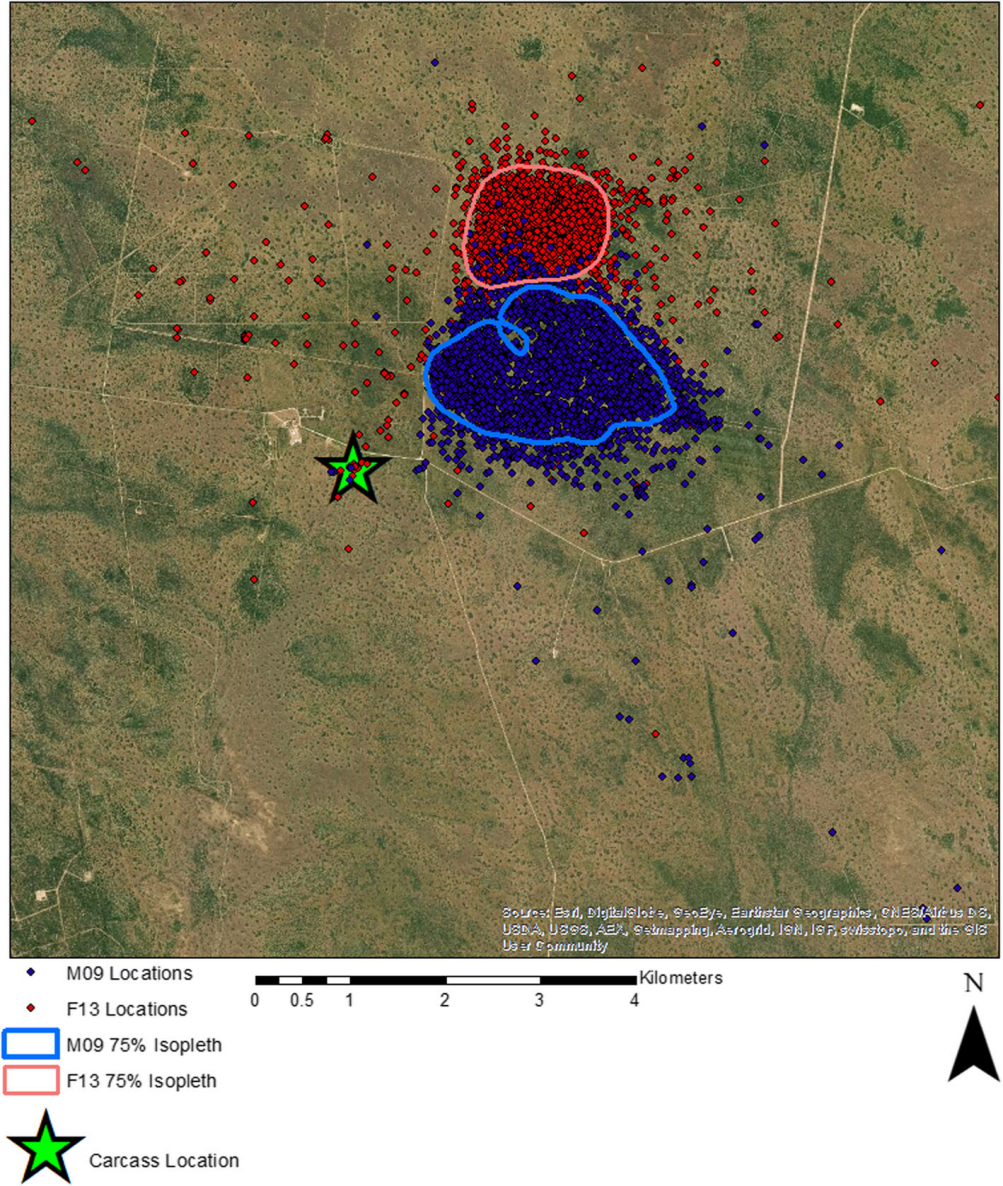
Territories of the 2 GPS-collared, coyotes M09 and F13, used in our example from the East Foundation’s San Antonio Viejo Ranch. Territories were delineated using the 75% isopleth of a fixed kernel density estimate of all locations for each individual. Note the location of the carrion resource near, but outside, both territories

We then used field data representing 2 coyotes to demonstrate the practical utility of our approach in describing the dynamics of animal space use (Fig. 1). We collected these data on the East Foundation’s 61,000 ha San Antonio Viejo Ranch (SAVR) in Jim Hogg and Starr counties in southern Texas. The East Foundation’s ranches are managed as a living laboratory to promote the advancement of land stewardship through ranching, science, and education. The area is dominated by shrub savannas, primarily composed of honey mesquite (*Prosopis glandulosa*), prickly pear (*Opuntia* spp.), cat-claw acacia (*Acacia greggii*), blackbrush (*Acacia rigidula*), whitebrush (*Alloysia gratissima*), and granjeño (*Celtis palida*), with early to mid-successional grasses, including three-awns (*Aristida* spp.), little bluestem (*Schizachyrium scoparium*) and windmill grasses (*Chloris* spp.).

**Fig. 2:**
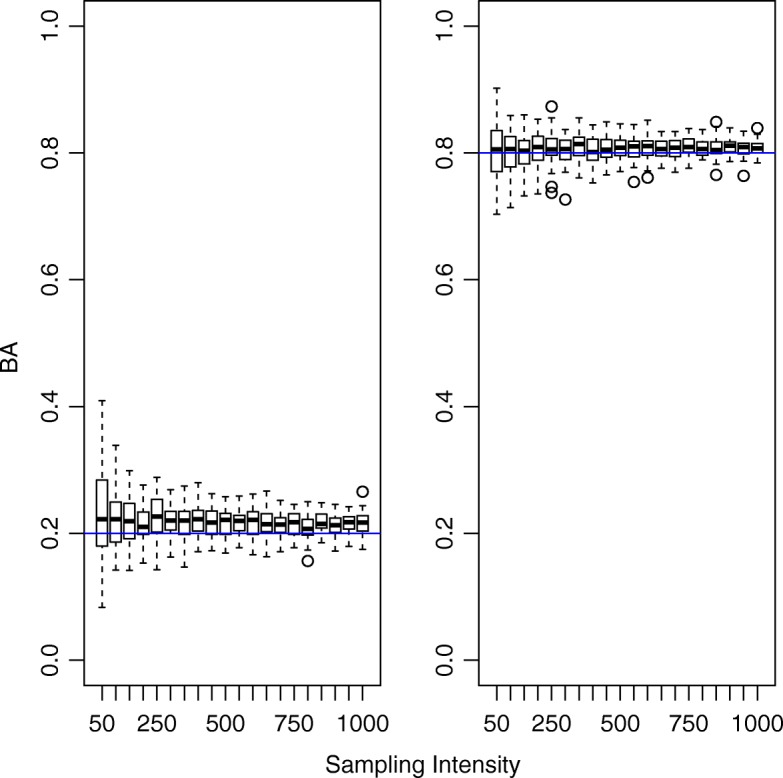
Distribution of estimated Bhattacharyya’s Affinity (BA) values as sampling intensity increases. Blue lines represent the true BA values of the parent utilization distributions

We captured individuals by helicopter using a net gun [34], fitted them with a Vertex Plus or Vertex Lite GPS collar (Vectronic Aerospace GmbH, Berlin), and released them at the site of capture on 10 December 2016 (n = 1) and 1 April 2017 (n = 1) as part of an ongoing study of coyote space use. These collars collected location data every 2 hours until 31 December 2017, when they automatically released from the animal. While our collars collected location data on identical schedules, this is not strictly necessary, as long as collars collect comparable numbers of locations over the same time windows. To standardize across collars, we omitted data prior to 1 April 2017 from the analyses presented below. Both coyotes were considered territorial [35], and occupied distinct, non-overlapping territories. A domestic cow (*Bos taurus x B. indicus*) died of unknown causes in an area well outside both territories (Fig. 1) during the week of 23 September 2017. Coyotes alter their patterns of space use to utilize carrion resources [36], so this event afforded us the opportunity to evaluate whether our methods would detect a change in spatial overlap between the coyotes in response to the presence of carrion.

We included time relative to death of the cow (before or after) as a dummy coded variable

7 $$ \begin{aligned} x_{t} \in \{0,1\} \\ x_{t} = \left\{\begin{array}{ll} 0,& \text{if}\,\, t < t_{carrion} \\ 1,& \text{if} \,\,t \geq t_{carrion} \end{array}\right\} \end{aligned}  $$

where *t*_*carrion*_ is the week of carrion deposition, to test whether that event had a persistent effect on the mean BA. Autocorrelation was modeled with *ARMA*(1,1) terms. This model is consistent with an interrupted time series design [37] and is analogous to an ANOVA for a beta-distributed variable with serial dependence. The resulting regression form consists of the marginal model

8 $$ \begin{aligned} BA_{t}|x_{t} \sim Beta(\mu_{t},\kappa_{t}) \\ logit(\mu_{t}) = x_{t}\beta_{1} + \beta_{0} \end{aligned}  $$

and copula

9 $$ \begin{aligned} \Phi(\epsilon_{t})\\ \epsilon_{t} \sim ARMA(1, 1) \end{aligned}  $$

Succinctly, this model tests for a persistent change in spatial interaction between 2 coyotes following the carrion deposition event, and estimates its magnitude.

## Results

### Simulation study

Our simulation showed that reasonably precise estimates of BA can be achieved with 150 sampled locations per time window at both high and low values of BA (*s*.*e*.=0.029; Fig. 2). Estimates based on as few as 50 relocations per window could be useful if the hypothesized effect of some covariate is sufficiently large. These results also suggest a slight positive bias at low BA values, which decreases with sampling intensity. At 50 locations per window, the average bias at a true BA of 0.20 was 0.0311 (*SE*= 0.00919), while at a true BA of 0.80 the average bias was -0.00077 (*SE*= 0.00641). The bias at low BA declined with increasing sampling intensity to 0.0155 (*SE*= 0.00253). The average bias at high true BA values never exceeded 0.0105 (*SE*= 0.00342).

**Fig. 3:**
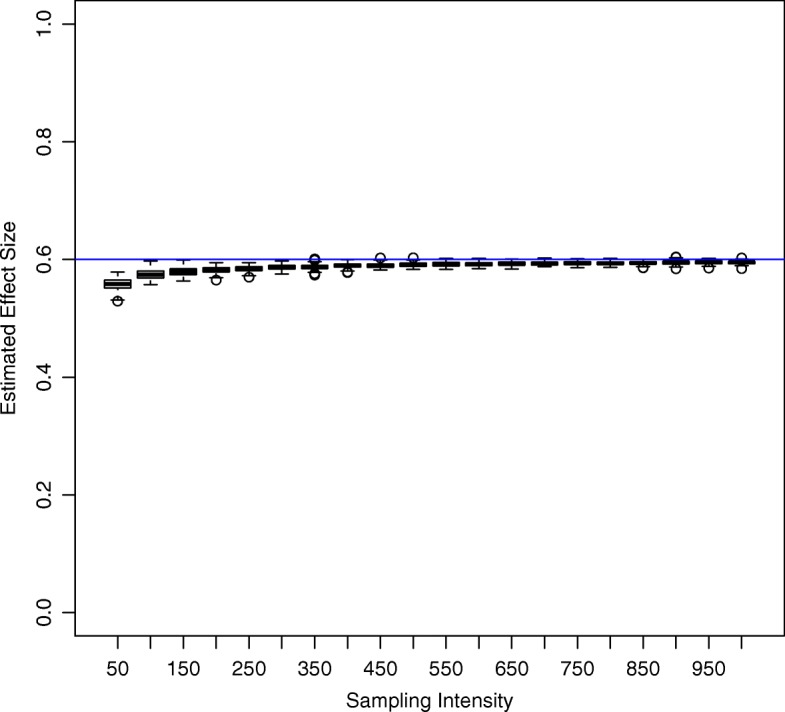
Estimated effect size of binary covariate on Bhattacharyya’s Affinity (BA) as a function of sampling intensity (sampled locations per time window). The blue line represents the true effect size

Parameter estimates from regression models stabilized quickly at 150 relocations, while error around the prediction slowly contracts beyond that point (Fig. 3). These estimates were slightly negatively biased, with an average bias of -0.0427 (*se*= 0.00106) at 50 locations/window, decreasing to a minimum of -0.00508 (*se*= 0.00106) as sampling intensity increased. This is likely due to the slight positive bias of low-valued BA estimates, which was strongly correlated with effect size bias across simulations (*r*= -0.784).

### Application to coyote data

**Fig. 4:**
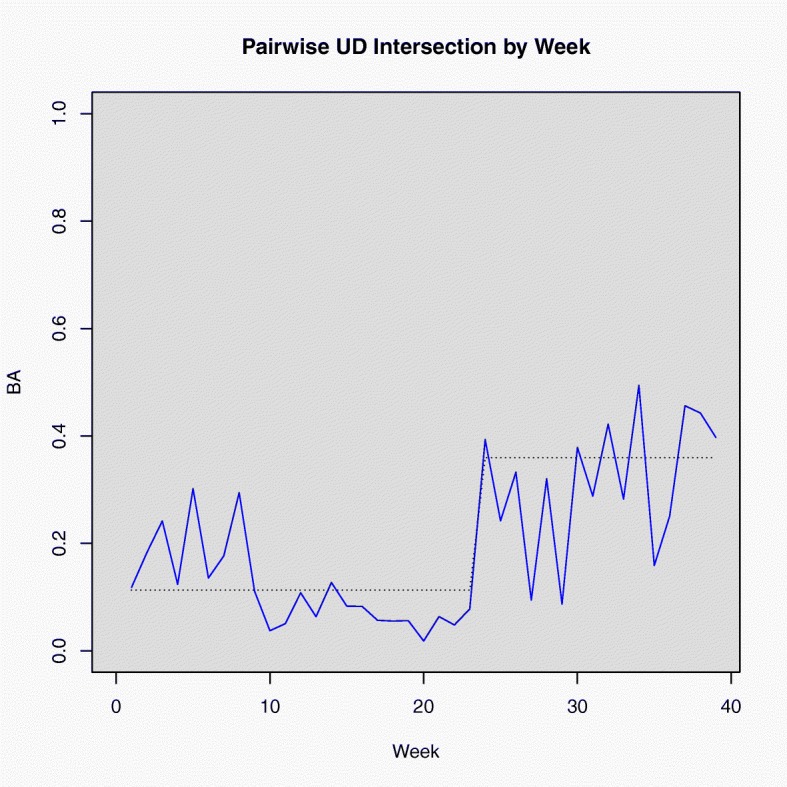
Time series of joint space use between the 2 GPS-collared coyotes from the East Foundation’s San Antonio Viejo Ranch, measured by Bhattacharyya’s Affinity (BA; blue line) and fitted values of the copula regression model (black, dashed line)

**Fig. 5:**
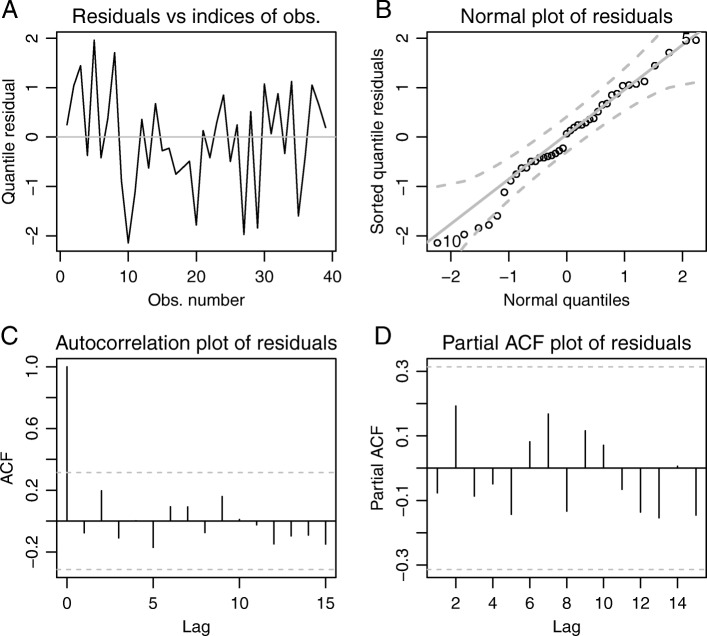
Residual diagnostics of beta regression model of two coyotes from the East Foundation’s San Antonio Viejo Ranch. **a** The plot of residuals through time shows an unaccounted for structural difference between weeks 0-9 and subsequent weeks, as well as potential outliers at weeks 20, 27, 29, and 36. **b** The Q-Q plot shows reasonable model performance, again suggesting possible outliers at weeks 20 and 36. **c**-**d** Autocorrelation and partial autocorrelation plots show no significant residual autocorrelation, meaning the *ARMA*(1,1) term adequately captured the dependence structure

The time series of BA values between the two coyotes indicated an obvious change in behavior following the appearance of the carrion resource (Fig. 4) and the beta regression model showed a significant effect of the carrion event (*P*<0.001; Fig. 4). The average UD intersection increased by 0.246, meaning that, on average, the 2 coyotes spent approximately 25% more time in the same places following the carrion deposition event. Upper and lower 95% CIs of this effect were 0.437 and 0.092, respectively. The graphs of observed and fitted values (Fig. 4), and the residuals (Fig. 5a) showed unaccounted structural differences between weeks 0–9 and weeks 10–24. Weeks 20, 27, 29, and 36 were identified as potential outliers (Fig. 5b), but overall the distributional form was appropriate. The *ARMA*(1,1) terms were significant (*P*<0.001 for both). Autocorrelation diagnostic plots supported the appropriateness of the assumed autocorrelation structure (Fig. 5c-d).

## Discussion

Our results are a proof of concept for the use of sequential measurements of UD intersections in a time series framework to capture dynamics of spatial interactions between 2 individuals. Results with simulated data reveal slight positive biases in low-valued BA estimates leading to slight negative biases in effect size estimates. However, the effect of such small biases on the ecological interpretation of results likely would be negligible in most cases. Further, sampling error is reasonable at achievable sample sizes with current GPS technology. Our framework is based on familiar analytic tools and results are readily interpretable. The framework also provides a much more detailed view of interactions through time compared to existing methods, as we demonstrated with the coyote example.

### Practical application and performance

Our methodology is applicable to a wide variety of ecological questions where there is an a priori hypothesis about the drivers of joint space use. Our coyote example focuses on the presence of a resource, however the imposition of some disturbance, management action, or life history events (e.g. breeding associated behavior) are equally well treated with our approach. Because our approach is couched in a regression context, continuous covariates are also valid, though beyond the scope of our simulations. These could include such variables as available forage, precipitation, or temperature extremes within time windows, or the researcher could include cosine transformations of time to evaluate seasonal effects, to name but a few. This allows considerable flexibility to address questions of joint space use.

The length of the temporal window over which UDs are estimated is a key consideration in applying this analysis. The appropriate choice will depend on the temporal scale of the motivating question and the ecology of the species. The length of time window must be matched to the scale of the phenomenon of interest. Specifically, the window must be fine enough to capture variation in joint space use attributable to the phenomenon [38]. Highly mobile animals, that change their patterns of space use often, may require shorter windows in order to capture relevant variation in joint space use than sedentary species. For example, cougars (*Puma concolor*) are known to exhibit frequent, recursive space use patterns [39], which would require short time windows relative to their return frequency to capture. The analysis may also be conducted with multiple window lengths to examine how overlap varies with temporal scale, allowing the researcher to identify when individuals partition space at fine temporal scales but overlap at larger ones. However, the finest temporal scale that can be considered is limited by the number of locations required to adequately estimate a UD.

Various authors have reported minimum numbers of locations required to obtain a reliable UD estimate with the methods we used [26, 29, 40]. Our simulations show acceptable results using a first-generation estimator with 150 samples per UD window and 100 windows, approximating hourly collection intervals over a 2-year period. This sampling regime is common for larger species [41–43], yielding 168 locations per week. This sampling intensity is sufficient to generate reliable UDs, given the inherently unbiased design of sampling at regular time intervals [26, 29], and gave adequate performance in our simulations. This sampling intensity is relatively easy to achieve for large species, but presently unattainable for smaller species incapable of carrying large batteries. These constraints may be alleviated by improvements in battery technology and efficiency of GPS collar circuits, as well as more efficient UD estimators.

The precision of BA estimates is a function of the performance of the KDE method used. While we utilized a first-generation estimator for simplicity and computational speed, any KDE method is suitable for this approach and the appropriate estimator will depend on the particular research question [16, 44]. Given that the true UDs in our simulations were bivariate normal, our use of the reference parameter is justified in the literature [25, 26]. However, this procedure is known to overestimate the 95% isopleth area of more complex UDs [26, 45, 46], suggesting that the density in the tails of the UD is overestimated. This may also be the case in our simulations, which would explain the greater degree of bias when the UDs intersect mainly in their tails (at low true BA values). This greater positive bias at low values would compress effect size estimates in cases when BA increased following disturbance, as in our simulations. On the other hand, if the effect was negative following the disturbance, its magnitude would be slightly overestimated. The magnitude of the bias is small in either case, as indicated at our lowest sampling intensity. A bias of 3% (our largest average bias) is unlikely to affect ecological interpretation of results, and may be safely considered negligible in most cases. More sophisticated methods may be less biased in the tails of the UD, reducing bias in parameter estimates. The relative performance of various KDE procedures within this context is an open question that warrants further research.

### Further development

Beyond technological improvements, there are analytical limitations to overcome to realize the full potential of our approach. Our techniques provide pair-level series, permitting analysis at the dyad level. Population level inference will require multivariate time series methodologies that accommodate potentially non-independent, beta-distributed response variables, which to our knowledge are currently unavailable. However, such methods do exist for short, non-stationary, Gaussian series that could serve as a conceptual basis for similar approaches with beta-distributed response variables [47]. Additionally, the approach we demonstrate here treats BA measurements as fixed values, though we show that they are estimated with error. Recent work provides a potential means to handle this source of error [9], and an appropriate hierarchical structure could be derived. Such development would be particularly important in sampling situations like our coyote example. Our simulation results suggest that sampling error of UDs at our bi-hourly schedule (84 locations/week) is appreciable at the lower BA values we observed between these individuals throughout the monitoring period (Figs. 2 and 4), thus the uncertainty of our parameter estimates may be particularly underestimated.

### Advantages of this approach

The residual analysis of the beta regression model of coyote interactions reveals an important advantage of our approach; there is another period of interaction early in the series that we have captured, but failed to explain (Fig. 5). This early period of interaction would have been masked in a simple analysis of UD intersections before and after the death of the cow, as would be done using previous methods. Assuming space use itself to be stationary over these time blocks is unwarranted. The time series framework we propose captures the nonstationary dynamics of space use patterns and provides a means to explain them. Additionally, our methodology yields a statistical test of the effect that until now was not possible. Although [9] produced a method to test the significance of a single BA estimate, our framework permits modeling the influence of 1 or more variables on the dynamics of joint space use in an interpretable way.

**Fig. 6:**
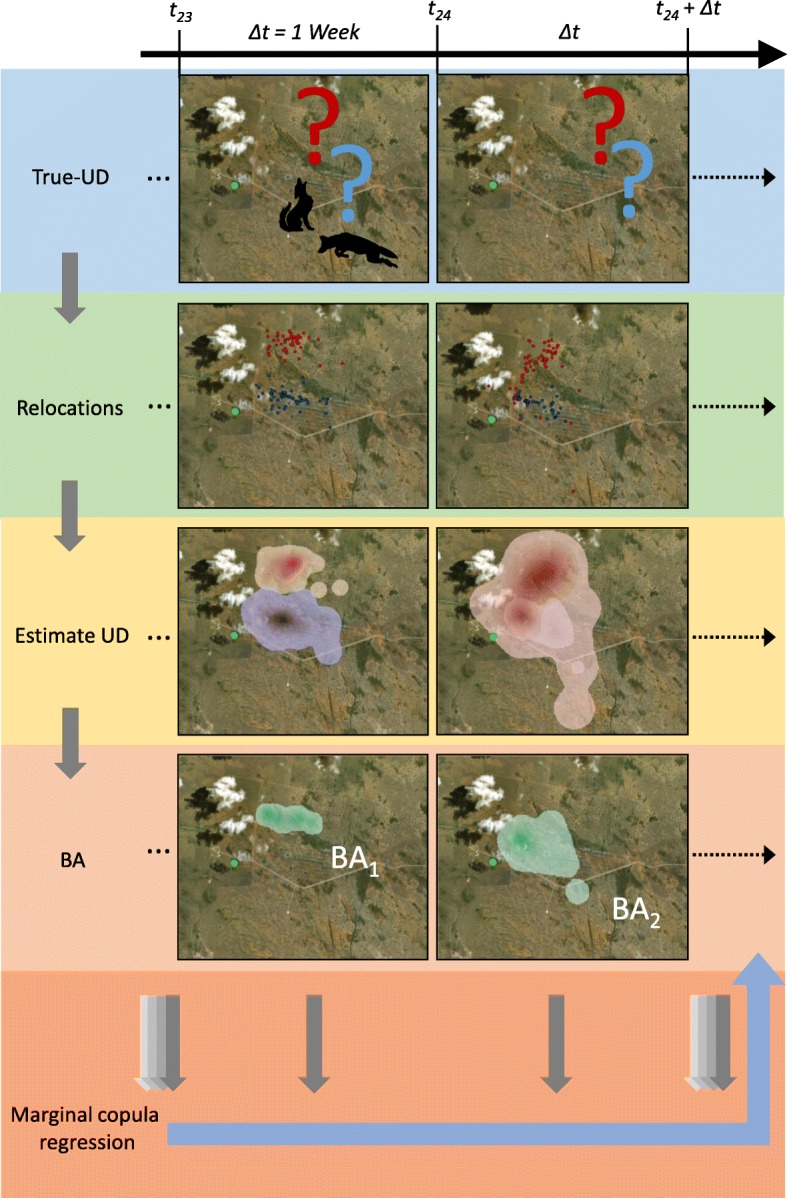
Visualization of the quantification of joint space use by the 2 coyotes from the East Foundation’s San Antonio Viejo Ranch during the week prior to the carrion deposition event (*t*_23_: carrion location marked with green dot) and during the week in which the event occurred (*t*_24_). Relocation data are analyzed to estimate the 2 individual space utilization distributions (UD; red dots and shading for the female, blue for the male), from which the joint UD volume is calculated (the integral of which is BA), which indicates the area of joint space use (green shading)

Each stage of our framework was selected for straightforward interpretability (Fig. 6). The probabilistic nature of UDs, and their widespread use by ecologists make them an attractive starting point. The intuitive interpretation of BA as a symmetric index of how much 2 individuals use the same space makes it a natural choice. More subtly, the choice of marginal copula regression over other appropriate time series methods also aids interpretability. The separation of the regression component from the correlated error structure allows straightforward interpretation of model coefficients, which is not possible with other available methods [24]. Despite the substantially different mathematical architecture, this means that interpretation of model coefficients is done in the same manner as GLMs, which are common in ecological literature. This familiarity makes our approach easily accessible to ecologists.

Fine scale dynamics, such as how movement trajectories change, or patterns in the distances between individuals could also be considered to examine inter-individual interactions [48, 49]. However, these approaches focus on fine-scale properties of movement, and answer related, but different questions [50]. Indeed, such analyses could serve as complimentary tools to our method. For example, joint space use may be used to examine similarity in habitat use, while information on the distances between individuals would provide information on how those individuals respond to each other at a finer scale (e.g. avoidance or attraction). Capturing these dynamics through time may elucidate mechanisms of resource partitioning between species.

The results of our approach are also readily visualized, which is of great heuristic value and lends intuitive context to the quantitative results. For example, we can visualize the change in joint space use by the 2 coyotes immediately before and after the carrion deposition event (Fig. 6). Mapping the UDs and the joint UD volume (the integral of which is BA) shows that joint space use before the event was concentrated along the boundary between the 2 territories. After the event, joint space use increased markedly as the female expanded her activity range toward the southeast, engulfing the activity range of the male, which also shifted slightly toward the southeast. Interestingly, both individuals moved synchronously away from the carrion initially, and did not converge on it until the following week, as confirmed by GPS locations converging at the carcass site (Fig. 1). The cause of these movements remains unknown, but their identification provides important contextual information that aids interpretation and the generation of ecologically-based hypotheses.

We argue that these properties also simplify communication of results to scientific peers and non-scientist stakeholders alike. The statement “on average, the 2 coyotes spent 25% more time in the same places each week after the carrion resource became available" is an accurate and meaningful interpretation of our results. An important caveat is that the individuals were not necessarily in those places at the same time within the week. Thus, the temporal grain and scale used in the analysis will affect interpretation. Nonetheless, such a statement carries implications for a variety of disciplines.

Finally, though we discuss linking joint space use to covariates selected for a priori hypotheses, other time series methods are applicable. For example, change detection methods allow researchers to segment time series into periods of similar behavior [51, 52]. These exploratory methods could be of great utility when periods of attraction or avoidance are expected, but when the time of their occurrence is not known. For example, some ungulates are known to partition space between sexes for most of the year, but aggregate during the breeding season [53]. Change detection methods could be used with a BA time series between sexes to objectively delineate when the breeding season occurs.

## Conclusions

This work represents a marked advance towards informative, tenable analysis linking variables to the dynamics of joint space use that is also communicable to non-scientists. This methodology has applications in many areas of applied ecology where the dynamics of animal interactions are of interest. Given limited time, money, and material resources, successful management requires focused efforts. Our methodology provides needed information that is intuitively understood by stakeholders. This facilitates effective communication between scientists and decision makers, ideally leading to efficient, spatio-temporally targeted management actions supported by valid analyses.

## Data Availability

The authors intend to archive the coyote data with MoveBank (https://www.movebank.org/)

## Abbreviations

ARMA: Autoregressive moving average
BA: Bhattacharya’s affinity
KDE: Kernel density estimate
SAVR: San Antonio Viejo Ranch
UD: Utilization distribution
